# Out of the Lab and Into the World: Analyses of Social Roles and Gender in Profiles of Scientists in *The New York Times* and *The Scientist*

**DOI:** 10.3389/fpsyg.2021.684777

**Published:** 2022-01-13

**Authors:** Tessa M. Benson-Greenwald, Mansi P. Joshi, Amanda B. Diekman

**Affiliations:** Department of Psychological and Brain Sciences, Indiana University Bloomington, Bloomington, IN, United States

**Keywords:** social roles, gender, media portrayals, science communication, communion, agency

## Abstract

Although representations of female scientists in the media have increased over time, stereotypical portrayals of science persist. In-depth, contemporary profiles of scientists’ roles have an opportunity to reflect or to challenge stereotypes of science and of gender. We employed content and linguistic analyses to examine whether publicly available profiles of scientists from *New York Times* and *The Scientist Magazine* support or challenge pervasive beliefs about science. Consistent with broader stereotypes of STEM fields, these portrayals focused more on agency than communality. However, profiles also challenged stereotypes through integrating communality, purpose, and growth. This analysis also found similar presence of communal and agentic constructs for both female and male scientists. The current findings highlight the importance of considering counterstereotypic representations of science in the media: Communicating messages to the public that challenge existing beliefs about the culture of science may be one path toward disrupting stereotypes that dissuade talented individuals from choosing science pathways.

## Introduction

In 1961, *Time* magazine’s Man of the Year Issue highlighting “United States Scientists” showed an array of scientists – each one male (Bath et al., 1961). Since then, the presence of women scientists portrayed in media has risen –in television shows (Orthia and Morgain, 2016), science textbooks (Pienta and Smith, 2012), magazines (Previs, 2016), and other mass media products (Steinke, 2013). Yet portrayals continue to present fewer women than men and often persist in emphasizing stereotypic notions of gender or science (Clark and Illman, 2006a; Chimba and Kitzinger, 2010; Steinke and Tavarez, 2018). A close analysis of media portrayals of the scientist role, and their potential impact, is thus warranted: Along with showing *who* a scientist is, media portrayals show the nature of scientific work, whether scientists meet and overcome challenges, and *why* scientists engage in their work.

The construction of science in specific media outlets is a cultural practice (Fürsich and Lester, 1996), and portrayals can both reflect and construct public understandings of science. Close analyses of the portrayals in the New York Times’ section “Science Times” offer insights into how science is constructed for broader audiences. For example, the topical coverage varies across time from 1980 to 2000, with shifting emphases on health/medicine relative to technology/engineering or natural/physical/life sciences (all of which are emphasized more than culture/history (Clark and Illman, 2006b). Further, engineering is rarely portrayed across this period (Clark and Illman, 2006a); when it was depicted, engineering was frequently portrayed as providing benefit to society and as a creative endeavor. However, female engineers were not represented during this period. Additionally, close analyses of portrayals in *The Straits Times*, a Singapore-based newspaper, demonstrate that the prevalence of health/medicine science is not purely specific to United States-based news outlets (Subramaniam, 2014).

Do these media portrayals reflect pervasive stereotypes about science? In the United States, stereotypes about STEM fields include perceptions that these careers offer opportunities for achievement, independent work, and competition (i.e., agency) but fail to offer opportunities to connect to others or to benefit society (i.e., communion; Stout et al., 2016; Diekman et al., 2017; Brown et al., 2018). The typical scientist is perceived to be less communal and more agentic than the typical man or woman (Carli et al., 2016). Physical appearance stereotypes also influence who is seen as a scientist: Women with more feminine facial features are seen as less suited for science careers (Banchefsky et al., 2016). Such stereotypes of science and scientists can dissuade people from engaging in science because the scientist role is perceived not to align with communality (Diekman et al., 2010). Here we examine how publicly available profiles of scientists support or challenge pervasive stereotypes about science.

### The Social Role of Scientist: What Is It and Why Does That Matter?

#### Specific and Diffuse Roles: Expectations of the Scientist and Gender Role

We adopt a social role framework (Eagly and Wood, 2011) to analyze the content of public portrayals of science (a) in terms of the specific scientist role and (b) in terms of whether role content varies by gender of the scientist. Social roles encompass beliefs about the behaviors linked to a particular social position (Biddle, 1986); some social roles are *specific* to particular contexts or duties (e.g., scientist), whereas some social roles are *diffuse* and exist across different contexts or duties (e.g., gender; Diekman and Schneider, 2010). For instance, women who are scientists contend with expectations about the female gender role and the scientist occupational role. Traditional gender roles associate women with communal traits oriented toward others and men with agentic traits oriented toward self-promotion. These communal and agentic trait expectations align with a traditional division of labor where women are primarily responsible for caregiving and men for leadership in public roles (Eagly and Wood, 2011). Modern gender role expectations of women include increased competence along with high communality due to women entering formerly male-dominated occupations and also maintaining domestic caregiving (Donnelly and Twenge, 2017; Eagly et al., 2019). This expanded view of expectations for women is reflected in the media. Media depictions of female athletes increasingly invoke portrayals of “powerful” that do not pit power against traditionally feminine traits such as concern for others (Bruce, 2016). For men, however, modern gender role expectations have remained relatively stable with an emphasis on agentic traits (Eagly et al., 2019). Thus, contemporary expectations for women include an emphasis on both communal and agentic characteristics whereas those for men emphasize primarily agentic characteristics, leading to expectations of gender similarity for agency.

Furthermore, gender differences can be minimized when men and women occupy the same specific occupational roles. Specific roles such as manager or subordinate can influence perceived dominance more than gender does (Moskowitz et al., 1994). Yet, when specific roles can be enacted in different ways, diffuse gender roles can continue to exert impact. For example, within the same physician role, women spend more time talking with patients than do men (Roter and Hall, 1998; Roter et al., 2002). Likewise, experience sampling data showed women across status hierarchies (e.g., boss, co-worker, and supervisee) displayed more communal behavior than did men (Moskowitz et al., 1994). A primary question in the current work, then, was along which dimensions gender differences in portrayals of science might emerge. Because contemporary gender roles differentiate men and women more strongly on communal than agentic attributes, we expected gender differences in portrayals of science to occur in communion more than in agency.

#### Media Portrayals of the Scientist Role

How do people learn about who scientists are and what they do? Beliefs about social roles derive from observing people within those roles (Koenig and Eagly, 2014). How media communicate about science shapes science beliefs for the public and within the scientific community (Steinke, 2005). Media communication unfolds as a cultural process wherein specific actors engage in interpreting and communicating knowledge (Lievrouw, 1990, 1992). For example, analyses of the “Science Times” section of *The New York Times* highlights the tendency for science journalists to uphold a stereotypic image of pristine science conducted mostly in a laboratory setting, while also challenging those images through attempts to humanize and demystify scientists (Fürsich and Lester, 1996). For instance, when engineering was mentioned, most profiles emphasized contributions to society (Clark and Illman, 2006a). Yet, not all coverage surrounding engineering and society is positive: National news coverage of robotics in surgery/medicine featured a negativity bias, highlighting the negative or unfavorable aspects of these surgeries (Ficko et al., 2017). Thus, the ways that science writers communicate can create and highlight images of science that uphold and challenge popular views of scientific knowledge and scientists.

From the perspective of psychological science, media portrayals are worthy of study as part of a larger set of cultural practices that can reflect and shape intrapersonal cognitions. For example, daily practices related to leisure, work, or family can both influence and be influenced by individual-level cognitions and motives (Markus and Kitayama, 2010). One example is that the content of magazine advertisements reinforces cultural norms of individualism vs. collectivism: Magazine advertisements in the United States focus on individual benefits and personal success, whereas advertisements in Korea focus on ingroup benefits and harmony (Han and Shavitt, 1994). Similarly, analysis of music preferences by social class reflected distinct models of agency, with middle/upper class agency emphasizing self-expression and working-class agency emphasizing resistance to pressure (Snibbe and Markus, 2005). Further, constructions of “gender equality” as reflected in children’s literature show that even books praised as non-sexist portrayed girls and women who adopt male-stereotypic attributes but not boys and men who adopt female-stereotypic attributes (Diekman and Murnen, 2004). In this way, representations of social groups in popular culture reflect and construct beliefs about those groups. Here, we employ archival methods to understand how the scientist role is communicated through media portrayals.

Some depictions might amplify gender stereotypes. For example, media representations of scientists emphasized women’s physical appearance but not men’s (Chimba and Kitzinger, 2010), reinforcing gender stereotypic emphases on women’s appearance. Similarly, publicly available profiles of scientists often simplify the female experience in science by overemphasizing the gender-specific challenges associated with being a woman in science (Mitchell and McKinnon, 2019). Indeed, profiles of female scientists tended to characterize them as a special set of individuals, highlighting personal-orientation toward familial roles, while profiles of male scientists focused more on their role as prominent scientists (Shachar, 2000). Such representations can highlight the overrepresentation of men in STEM and reinforce perceptions of the chilly climate of STEM for women. Experimental research demonstrates the impact of media representations: Women who read a newspaper article that described computer scientists as confirming the “geek” stereotype were less interested in computer science careers than those who read an article that challenged computer science-“geek” stereotypes (Cheryan et al., 2013). In the current research, we asked how the scientist role is portrayed and how this intersects with gender roles.

Analyzing print media portrayals of scientists from a social role perspective can provide insight into the features of the scientist role that are highlighted, and whether these roles are differentially portrayed for male and female scientists. In the current work, we explored whether the scientist role is portrayed similarly or differently for male and female scientists.

### Beliefs About Science Roles: Why and How Do Scientists Engage in Science?

Portrayals of scientists in the media not only depict who is a scientist but the specific behaviors and motivations of the scientist role. These portrayals can inform people’s stereotypes of scientists – providing them with information on how and why scientists pursue their work. In the current research, we examined portrayals of scientists to determine whether content aligns with these dimensions of the scientist stereotype. What is the motivation behind scientists’ research questions? How do people become successful scientists?

#### Beliefs About Scientists’ Purpose

One way to challenge stereotypes of science as lacking communality is to connect scientific work to a broader purpose: Science conducted in the lab matters for the world, but stereotypes of science can neglect this broader impact (e.g., Diekman et al., 2020). Here, we examine whether and how profiles of scientists portray the purpose of pursuing scientific work. If media representations do portray scientists’ purpose, do these overarching purposes reflect agency (e.g., attaining success or recognition) or communion (e.g., benefiting society)? Investigating scientific work in terms of a broader purpose beyond the self can confer motivational benefits. For instance, students who considered the broader purpose of their academic work persisted and succeeded more than students who did not, in both laboratory or longitudinal studies (Yeager et al., 2014). Yet, it is unclear whether media depicts scientists as pursuing their work for broader purposes, and thus this research provides an initial answer to that question.

We also anticipate the portrayal of scientific work as communal or agentic to vary by how gender stereotypes are amplified among female and male scientists. How the gender role is portrayed in science may also influence the perceived purpose of the scientist role. If profiles reflect contemporary gender stereotypes, they may connect the scientific work of female scientists to a communal purpose more than for the work of male scientists. However, if the scientist role takes prominence over the diffuse gender role, we would expect female and male scientists to discuss either type of purpose at similar rates. Thus, the perceived purpose of the scientist role is likely to vary based on how the gender role is emphasized in portrayals.

#### Beliefs About Scientific Success and Struggle

Another set of pervasive beliefs about science focuses on how success as a scientist is characterized: Is success marked by the illustrious performance that aligns with innate talent or marked by an effortful, ongoing process where capacities are developed over time? Stereotypically, scientific fields are thought to require innate brilliance, particularly in more male-dominated fields such as physics (Leslie et al., 2015). Female STEM students think they need to work harder than their peers do, and this perception of differential effort expenditure negatively predicts belonging in STEM (Smith et al., 2013). The belief that capacity in science is largely innate can reflect a fixed mindset that emphasizes performance and display of success, whereas the belief that capacity in science is developed through challenges and effort can reflect a growth mindset (Dweck, 1999). In all, a dominant narrative of scientific success is that it is a product achieved through innate talents, rather than an effortful and ongoing process.

Clear evidence exists that disrupting pervasive narratives of success can shift cognitions and behaviors. For example, potential student members highlighted their effort more for clubs emphasizing a growth mindset rather than a fixed mindset (Murphy and Dweck, 2010). In workplace settings, women who considered working at a growth-oriented company report fewer concerns about being stereotyped and respond more constructively to negative feedback than those who considered working at a fixed-oriented company (Emerson and Murphy, 2015). Further, perceiving people in STEM environments and careers as holding fixed beliefs about math ability negatively predict women’s sense of belonging (Good et al., 2012) and more strongly predict interest in science careers than gender stereotype beliefs (Barth et al., 2018). Representing success in STEM fields as resulting from effort, rather than a product of innate ability, can foster motivation among women in particular (Smith et al., 2013). Given this evidence, a key question for the current research is whether media profiles of successful scientists acknowledge the efforts and challenges involved in the process of success or whether they reify stereotypes of the “brilliant scientist.”

Further, attributions for overcoming struggles can either confirm or disconfirm stereotypes. A scientist can acknowledge challenges but portray overcoming them as a solo endeavor, which would support stereotypes about STEM as independent. In contrast, a scientist might acknowledge challenges and portray overcoming them with the help of mentors, peers, or family – such a portrayal would provide a counterstereotypic image of STEM.

### Current Research

We investigated how the scientist role is portrayed in elite publications. This study employed content and linguistic analyses to examine whether publicly available, in-depth profiles of contemporary scientists support or challenge pervasive beliefs about science. In line with past research, we expected portrayals to reflect stereotypic beliefs about science, in emphasizing agency over communion (Hypothesis 1) and discussing the purpose of scientific research as agentic rather than communal (Hypothesis 2). Further, we anticipated that portrayals would show success as a product of performance due to innate talent rather than an effortful, iterative process (Hypothesis 3).

We examined competing hypotheses about the gendered portrayals of scientists (Hypothesis 4). If the diffuse gender role takes precedence, then portrayals of female and male scientists will be more different than similar. Because contemporary gender roles differentiate men and women more strongly on communal than agentic attributes, we expected portrayals of female scientists to emphasize communion more than those of male scientists. If the specific social role (i.e., scientist) takes precedence, then portrayals of female and male scientists will be more similar than different.

## Materials and Methods

### Materials

We retrieved scientist profiles from two United States-based publications, *The New York Times* and *The Scientist Magazine*, that provide publicly available, in-depth profiles of scientists. The depth of these portrayals allowed for a more nuanced analysis than would have been possible with other media sources (e.g., news broadcasts). *The New York Times* (NYT) is a daily newspaper aimed at a wide readership; its audience tends to be younger, better educated, and of higher income than the average American adult (Pew Research Center, 2012). *The Scientist Magazine* (TS) is a monthly magazine and website aimed at life science researchers, the majority of whom work in academia (42.4%) or industry (38.9%; The Scientist, 2021). These different readerships allow us to investigate media portrayals aimed at varying audiences. The NYT includes a broad public readership, which positions it to have an impact on perceptions of the scientist role to shape beliefs. Profiles from TS focus on individuals within the life science track; they add to this research because they are also publicly available, similar in length and detail to NYT profiles, but offer a much larger sample size. Additionally, because TS has an audience primarily made up of STEM professionals, these profiles have the potential to influence the beliefs and norms that scientists *themselves* hold about the discipline. Analyzing the two publications together thus allowed the detection of robust patterns across different outlets.

Because our interest was in contemporary depictions of scientists, we located online profiles of scientists in these publications published since 2011 (when the NYT began its *Profiles in Science* series and the TS began its *Profiles* series in their current forms). For both sources, profile publication dates ranged from 2011 through 2019. To compare across profiles, we only coded profiles presenting a single scientist (this resulted in the exclusion of two NYT articles that included two scientists within one profile). The resulting data set included 27 profiles from NYT (11 women; 16 men) and 97 profiles from TS (39 women; 58 men).

### Text Analysis

Each set of profiles was analyzed with Linguistic Inquiry and Word Count (LIWC; Pennebaker et al., 2015a). LIWC reports the proportion of text that includes terms from pre-established specific dictionaries that capture specific constructs based on conceptual categories (e.g., positive emotions). We analyzed text using previously validated and reliable dictionaries of agency and communion (Pietraszkiewicz et al., 2019). Example communal dictionary items included *collaboration* and *altruism* and example agentic dictionary items included *achievement* and *autonomous*. The procedure for developing these dictionaries followed protocol detailed in the official LIWC2015 psychometrics and development manual (see Pennebaker et al., 2015b for more details). The developers rigorously tested convergent and discriminant validity of these dictionaries with other language-based measures of psychology using Latent Semantic Analysis (see Pietraszkiewicz et al., 2019 for more details).

### Content Analysis

Two trained independent coders, who were blind to hypotheses, coded the profiles. All codes were dichotomous (presence or absence) to increase reliability and to follow prior research (e.g., Cheryan et al., 2013, Study 1). The coding scheme was determined prior to coding or data analysis. Categories and descriptions are given in Table 1. Interrater reliability was calculated using Cohen’s kappa and disagreements were resolved through discussion.

**TABLE 1:** Coding categories and descriptions.

Category	Elements
*Purpose*	Articulate motivation for scientific pursuit; Why are they pursuing this research/did they become scientists?
*Communal purpose*	Helping others, serving humanity, serving community, working with people, connection with others, attending to others, caring for others, mentoring, and teaching
*Agentic purpose*	Power, recognition, achievement, mastery, self-promotion, independence, individualism, status, focus on the self, success, financial rewards, self-direction, demonstrating skill or competence, and competition
*Success*	Discuss success/being successful; particular aspects of success
*Continuing effort*	Ongoing process; talk about future directions, questions, and goals
*Already achieved*	Completed, behind them, or already achieved
*Struggle*	Acknowledge difficulties in their career; e.g., challenges with education (graduate school), career life, or research (failed studies, rejected manuscripts)
***Overcoming struggle***	
*Other attribution*	Overcoming challenge due to mentors, teamwork, or other people
*Self attribution*	Overcoming challenge due to own actions or characteristics, e.g., hard work

*Purpose type (communal, agentic) and overcoming struggle (other attribution, self attribution) were not mutually exclusive; profiles could be coded as mentioning both, either, or none. Success categories were mutually exclusive (either continuing effort or already achieved).*

#### Communal and Agentic Purpose

Coders identified whether the scientists mentioned an overarching purpose of their work or not (NYT, *k* = 0.78, 96.3% agreement; TS, *k* = 0.73, 93.8% agreement) using a coding scheme developed from previous literature (Yeager et al., 2014). Coders then noted whether the purpose was communal or not (e.g., working together, helping others; NYT, *k* = 0.87, 96.3% agreement; TS, *k* = 0.85, 92.8% agreement), and whether the purpose was agentic or not (e.g., independence, control over own work, financial rewards; NYT, *k* = 0.85, 92.6% agreement; TS, *k* = 0.74, 90.7% agreement). As a result of the combination of communal and agentic codes, profiles could be only communal, only agentic, both communal and agentic, or neither communal nor agentic. Communal and agentic purpose codes were developed by drawing on the goal congruity literature (Diekman et al., 2010, 2020).

#### Beliefs About Success

Aspects of success were coded using a scheme developed on the basis of prior literature on malleability of intelligence (Blackwell et al., 2007) and ability beliefs (Leslie et al., 2015). Coders noted whether or not the scientists discussed their successes (NYT, *k* = 0.78, 96.3% agreement; TS, *k* = 0.75, 96.9% agreement), and whether discussion of success was coded as either achieved or as a continued effort (NYT, *k* = 0.86, 92.6% agreement; TS, *k* = 0.76, 92.8% agreement).

Coders noted whether the scientist discussed overcoming a struggle (present or absent; NYT, *k* = 0.81, 92.6% agreement; TS, *k* = 0.77, 89.7% agreement) using a coding scheme drawing on the role of effort (Smith et al., 2013). Examples included difficulty getting experiments to work or challenges handling graduate school or early career life. Coders identified whether scientists attributed overcoming struggles to themselves (e.g., working hard and pushing themselves through it; present or absent; NYT, *k* = 0.85, 92.6% agreement; TS, *k* = 0.89, 94.8% agreement) or others (e.g., working closely with mentors and being involved in teamwork; present or absent; NYT, *k* = 0.82, 92.6% agreement; TS, *k* = 0.83, 93.8% agreement).

## Results

Given the different nature and sample sizes of the publication outlets, we report results separately by outlet. Despite different readerships, both outlets show largely similar findings. For robustness, we focus on patterns that emerge in both the NYT and TS. First, we use LIWC text analysis to test whether profiles reflect a stereotypical focus on agency (Hypothesis 1) through a higher frequency of agentic words, relative to communal words. Next, we use results from trained coders and qualitative examples to elaborate the LIWC analyses and to document specific depictions of scientist roles. Here, we investigate whether profiles highlighted an agentic rather than communal purpose of scientific research (Hypothesis 2) and focused more on success as achieved rather than developed (Hypothesis 3). For each of these questions, we tested whether or not profiles of female and male scientists differed in their content (Hypotheses 4). As moderation hypotheses, these analyses are presented alongside tests for Hypotheses 1, 2, and 3.

### Do Profiles Reflect a Stereotypical Focus on Agency?

**FIGURE 1:**
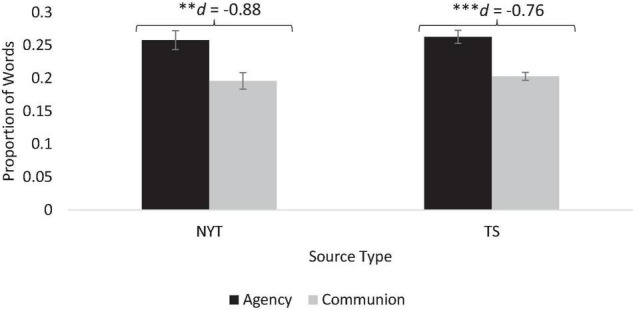
Scientist profiles include more agentic than communal words. Proportion of agency and communion words used with each profile source. Proportion of words is based on LIWC calculations and output (Pennebaker et al., 2015b). ^∗∗^*p* < 0.01, ^∗∗∗^*p* < 0.001.

To determine whether these profiles reflected stereotypic beliefs about science, we tested whether the LIWC text proportions included higher frequencies of agentic words than communal words, and whether this effect was moderated by scientist gender. A 2 (focus: agentic, communal) × 2 (scientist gender: female, male) mixed model analysis of variance, with focus as a within-subjects factor, revealed only a significant main effect of focus [NYT: *F*(1,25) = 8.90, *p* = 0.006, η^2^*_*p*_* = 0.26; TS: *F*(1,95) = 27.43, *p* < 0.001, η^2^*_*p*_* = 0.22]. Profiles included significantly more agentic than communal terms, supporting Hypothesis 1 (Figure 1). The main effect of scientist gender and the Focus × Gender interaction did not attain significance, *p*s > 0.60, consistent with the gender-similarity direction of Hypothesis 4.

#### Purpose: Integrating Communion and Agency

In both publications, content analyses revealed that the vast majority of profiles mentioned purpose (NYT: 100.0%; TS: 83.5%). Our Hypothesis 2 prediction was that profiles would highlight agentic more than communal purpose, but the observed data pattern was more nuanced. Scientists did frequently discuss agentic purpose, but they also frequently discussed communal purpose. Many scientists described the purpose of their work as reflecting both communal and agentic values (NYT: 44.0%; TS: 40.2%), though others focused solely on communal values (NYT: 41.0%; TS: 4.0%) or solely on agentic values (NYT: 7.0%; TS: 51.5%). Example statements of communal and agentic purpose are given in Table 2.^1^ Overall, the presence of both communal and agentic purpose demonstrates that scientific work can be portrayed as including communality. Indeed, these profiles provide a route through which media depictions might easily highlight the presence of both agency and communality in the scientific profession – through the prominent discussion of purpose for work.

**TABLE 2:** Selected examples of communal and agentic purpose.

Purpose	Quote	Source
*Communal*	We trust everyone and we share. There will be people who take advantage, but there have only been a few of those. So I learned … to give everyone maximum trust and then change this strategy only if they fail that trust. We collaborate easily because we give out everything and we also easily get reagents and tools that we may need	Azvolinsky, 2016
*Agentic*	It was a golden opportunity because it would leave me with plenty of time and resources to do what I wanted to do without worrying about getting grants or being subject to supervision	Azvolinsky, 2015
*Integrated communal and agentic*	I hope there is now a sustaining culture of scientists helping each other and keeping their eye on changing the world. That’s the goal. Being first author on the manuscript is not the goal. The goal is to change the world	Scudellari, 2013a

Tests of scientist gender as a moderator (Hypothesis 4) did not detect a difference between men’s and women’s profiles in the portrayal of scientific work as communal, agentic, or both [NYT: Breslow-Day χ^2^ (1, *N* = 27) = 1.07, *p* = 0.30; TS: Breslow-Day χ^2^ (1, *N* = 97) = 1.96, *p* = 0.16].

### Do Profiles Reflect Stereotypical Beliefs About Scientific Success?

In both publications, scientists frequently discussed their success (NYT: 100.0%; TS: 87.2%). Surprisingly, scientists frequently described pursuing success as an ongoing process (NYT: 51.9%; TS: 83.5%) rather than already achieved (NYT: 44.4%; TS: 16.5%); significant difference within TS, *Z* = 9.33, *p* < 0.001. For example, one scientist said, “You start down a path… you try to be creative and curious and figure things out, but you don’t know where it’s going to go” (Vence, 2015). This emphasis on the continuing pursuit of success was contrary to pervasive stereotypes that do not emphasize growth and effort (Hypothesis 3).

**FIGURE 2:**
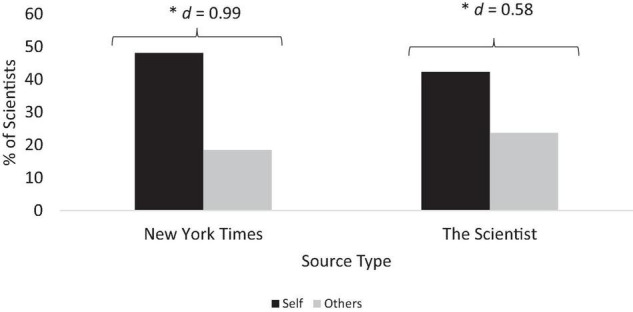
Attributions of overcoming struggles to the self more than to others. Percentages of scientists who attributed overcoming struggles to self or others. ^∗^*p* < 0.05. *New York Times N* = 27; *The Scientist N* = 97.

Scientists also frequently discussed overcoming struggles (NYT: 63.0%; TS: 67.0%), contrary to Hypothesis 3. Not surprisingly, many scientists attributed overcoming struggles to their personal qualities and effort (NYT: 48.1%; TS: 42.3%). Yet, a sizeable minority of scientists explicitly credited other people as essential in helping them overcome struggles (NYT: 18.5%, TS: 23.7%). Self-focused attributions were more prevalent than other-focused attributions in both outlets (Figure 2; NYT: *Z* = 2.31, *p* = 0.02; TS: *Z* = 2.75, *p* = 0.006). Demonstrating self-focused attributions for success, one scientist’s mantra is “If I can do it, so can you” (Broad, 2014). Demonstrating other-focused attributions, one scientist discussed her mentor’s help in developing her ability to formulate and test a question while emphasizing that “the answer wasn’t the important part” (Scudellari, 2013b). Overall, depictions of success – even among these prominent scientists – reflected persevering through challenges.

We also tested for scientist gender differences in discussion of struggles (Hypothesis 4). Men (NYT: 62.5%; TS: 62.1%] and women (NYT: 63.4%; TS: 74.3%) did not statistically differ in their discussion of struggles [NYT: χ^2^ (1, *N* = 27) = 0.004, *p* = 0.95; TS: χ^2^ (1, *N* = 97) = 1.59, *p* = 0.21] or their attributions for overcoming these struggles [*Self*: NYT: χ^2^ (1, *N* = 27) = 1.03, *p* = 0.21; TS: χ^2^ (1, *N* = 97) = 2.17, *p* = 0.14); *Others*: NYT: χ^2^ (1, *N* = 27) = 0.001, *p* = 0.97; TS: χ^2^ (1, *N* = 97) = 0.73, *p* = 0.39].

## Discussion

The current research illustrates that in-depth, contemporary portrayals of the scientist role both reflect and challenge stereotypes of science. Pervasive beliefs about science were reflected in text analyses documenting greater frequencies of agentic than communal words, supporting Hypothesis 1 and providing additional evidence of stereotypes of scientists and scientific work as focused more on agency than communality (Diekman et al., 2010; Carli et al., 2016).

Yet pervasive beliefs about science were also challenged in these profiles. The prediction that scientists would link their scientific pursuits to agentic but not communal purpose (Hypothesis 2) was only partially upheld: Scientists did link their work to agentic values, but they also explicitly linked their work to communal values. Further, contrary to Hypothesis 3, many scientists described their success in terms of continuing to work through challenges. Thus, the science portrayed in these profiles may serve as a model for how to describe science in ways that do not reify stereotypes of STEM fields as lacking in communality and requiring innate success. Content of profiles did not differ by scientist gender: Profiles of female and male scientists included similar frequencies of agentic terms and communal terms. These patterns suggest that although the specific scientist role highlights agentic aspects, the scientist role is portrayed as including communality for both men and women (supporting the gender-similarity version of Hypothesis 4). These findings thus contribute to understanding the intersection of role stereotypes and gender stereotypes (Eagly and Wood, 2011) by exploring how public-facing media communicates not just *who* is in the role but *how* it is enacted.

### Theoretical Contributions

The present research contributes to the understanding of role stereotypes and gender stereotypes (i.e., social role theory; Eagly and Wood, 2011) by exploring how public-facing media communicate the goal opportunities of scientific pursuits, and whether aspects of scientific pursuits are portrayed differently for men and women. Pervasive stereotypes hold that careers in science lack communality (Diekman et al., 2011, 2017) and that scientists attain success with little effort (Smith et al., 2013) and much innate talent (Good et al., 2012; Leslie et al., 2015). In the profiles analyzed here, scientists integrated *both* agency and communality into their scientific work, discussed whether and how they overcome challenges, and provided the overarching purposes for engagement with science. In this way, these profiles modeled both agentic and communal behaviors in science. The explicit integration of communal activities and purpose in science, alongside agentic ones, can shift beliefs about the goals that science affords, and these cues are interpreted similarly whether the scientist is a man or woman (Clark et al., 2016; Fuesting and Diekman, 2017). Thus, the current study advances understanding of the interplay between role and gender stereotypes by illustrating how naturalistically-occurring profiles of scientists integrate agentic and communal aspects of science. As such, the current work joins existing literature to demonstrate the possibility of integrating both agentic and communal aspects in portrayals of scientists’ work.

Analysis of these in-depth profiles allowed for a close examination of the interplay between the specific social role of scientist and diffuse gender roles. The finding that female and male scientists similarly integrated agency and communion provides support for the social role theory principle that gender differences may be constrained by the career role. This finding aligns with prior work examining profiles from just *The New York Times* (Mitchell and McKinnon, 2019), in which communal and agentic characteristics were attributed to both female and male scientists. As women enter and advance in science roles, they may introduce communally-oriented purpose and activity to a greater extent, similar to evidence that legislative bodies with greater proportions of women are more likely to introduce legislation focusing on education and health care (Swers, 2013). Yet, the recruitment of communally-oriented men into these roles also has the potential to disrupt public perceptions of STEM as lacking communality (Boucher et al., 2017). The framework adopted here can thus provide insight into how social roles shape gender, and how gender shapes social roles.

### Practical Implications

Meeting the demands of contemporary society requires in-depth investment in the science, technology, engineering, and mathematics (STEM) workforce. Despite the perks of careers in STEM (i.e., lower unemployment rates and higher salaries relative to other fields; McFarland et al., 2018), the demand for workers far exceeds the available supply in the United States (National Science Board, 2018). In addressing this problem, the cultural image of science serves as both a challenge and an opportunity (Bybee, 2010). The challenge is that stereotypic messages about STEM culture can dissuade talented individuals from entering STEM careers. Yet, the opportunity is that the dominant cultural image can be challenged through messages that integrate counterstereotypic elements. Indeed, framing political careers as serving the community increased women’s positivity toward entering political leadership (Schneider et al., 2016). This work thus has implications for science educators, media practitioners, and content creators: Depictions of scientists in public communications and popular culture provide an avenue to portray lesser known aspects of science by highlighting opportunities for collaboration, success through effort, and humanitarian purpose. Indeed, emphasizing opportunities for collaboration and humanitarian purpose has important implications for public trust in science: Scientific domains that were described as prosocially-oriented (vs. power-oriented) were trusted more and perceived as a higher funding priority (Benson-Greenwald et al., 2021b).

### Limitations and Future Directions

A primary limitation is that the focused nature of this content analysis included two publications, both based in the United States, which limits generalizability to other public-facing media and other countries. We chose these outlets because the depth of these portrayals allowed for a more nuanced analysis than would have been possible with other media sources (e.g., news broadcasts). We also chose two publications that serve different audiences and missions: Although both are publicly available, *The New York Times* addresses a broader populace that is better-educated, younger, higher-income adult than the average American (Pew Research Center, 2012), whereas *The Scientist* addresses people in the life sciences (The Scientist, 2021). These different readerships are a strength given that the goal of the research was to understand depictions of science and scientists in contemporary culture. As highly regarded, prominent publications, these two outlets might be on the front lines of challenging pervasive STEM stereotypes, serving as a model for other written public communications of science. Nonetheless, documenting how scientist and gender roles intersect in other forms of media and across countries is necessary.

The content analysis methods employed here can document patterns but cannot speak to the source of these patterns. We note that the content of these profiles may stem from the scientists, the questions asked by interviewers, or from editorial decisions about what to cut and what to keep: Multiple contributors may prevent or perpetuate stereotypes appearing in print. For example, the specialized audience of these publications may have an influence on how decision makers at *NYT* and *TS* depict these scientists. Different media can emphasize different aspects of science, and these variations have implications for resulting beliefs. Future work could analyze the questions interviewers pose to scientists, editors’ comments on articles, and direct transcripts of these interviews to understand more precisely processes that contribute to the end-result portrayals analyzed in this research.

We note that these publications did not consistently provide information about the ethnicity of the featured scientists. The current representations limit the study of intersectionality among gender and other identities, and how intersectional identities inform the enactment and portrayal of science. Because shared racial and gender identity is key in promoting positivity toward science among Black female students (Pietri et al., 2020), presentations that signal such shared identity clearly may be more impactful. The study of portrayals of science in media is important because these representations can encourage a broad array of talented individuals to enter science careers, broadening the mold of who participates in scientific work.

The current research documented the content of these scientific portrayals but did not provide evidence that these portrayals shift readers’ beliefs. Prior research provides strong evidence for this process (Brescoll and LaFrance, 2011; Cheryan et al., 2013), but documenting the impact of these naturalistically-occurring portrayals is an important next step. Indeed, work in our laboratory has found that participants who read a scientist profile that incorporated communal purpose and overcoming difficulties, relative to a profile that focused only explaining research, fostered more positive attitudes toward science careers (Benson-Greenwald et al., 2021a). This enhanced positivity was due to the perceived availability of communal opportunities in science, rather than to perceived agentic opportunities in science. Because beliefs about goal opportunities and success can deter underrepresented groups in science (Smith et al., 2014), such research could inform interventions targeted toward broadening participation in STEM.

Beyond integrating both communal and agentic content, many of these profiles discussed communal and agentic purpose at different levels of action. For example, the scientist quoted as saying “The goal is to change the world” expresses being an agent that acts on the world from a focus on others which differs from the self as an independent agent, such as the scientist who says they wanted “to do what I wanted to do.” These examples highlight that agentic or communal purposes might be enacted in more individual vs. collective ways (Triandis, 1989). Particularly important to note is that the content of purpose as agentic or communal is only one dimension of describing the properties of purpose; others might include whether the purpose engages independent or interdependent aspects of self (Markus and Kitayama, 1991), or abstract or concrete construals (Steinberg and Diekman, 2018). Concrete construals focus on the local, specific properties, whereas abstract construals focus on general, global properties (Trope and Liberman, 2010). Perceptions that STEM careers afford fewer opportunities for communion than agency occur at concrete construals but not at abstract construals (Steinberg and Diekman, 2018). Thus, an interesting direction for future work could be to document not only the communal or agentic content of purpose but also other properties that might have psychological consequences.

## Conclusion

Media representations offer the ability to portray the world not only as it is but also as it could be. Media portrayals of scientists and scientific work are essential to understand because they offer glimpses into roles that people may otherwise not experience. The current analysis found that profiles of scientists communicate messages that both support and disrupt stereotypes about science. Such profiles provide information about not only who is in the role, but also about the culture of science. Communicating messages to the public that challenge existing beliefs about the culture of science may be one path toward challenging pervasive and problematic stereotypes that often dissuade talented individuals from choosing science careers.

## Data Availability Statement

The raw data supporting the conclusions of this article will be made available by the authors, without undue reservation.

## Ethics Statement

The studies involving human participants were reviewed and approved by Indiana University, Institutional Review Board. The patients/participants provided their written informed consent to participate in this study.

## Author Contributions

TB-G developed the research topic and study design, performed statistical analyses, led the writing, and revised the manuscript. MJ and AD contributed to the conception and design of the study and contributed substantively to the writing and revision of the manuscript. All authors contributed to the article and approved the submitted version.

## Conflict of Interest

The authors declare that the research was conducted in the absence of any commercial or financial relationships that could be construed as a potential conflict of interest.

## Publisher’s Note

All claims expressed in this article are solely those of the authors and do not necessarily represent those of their affiliated organizations, or those of the publisher, the editors and the reviewers. Any product that may be evaluated in this article, or claim that may be made by its manufacturer, is not guaranteed or endorsed by the publisher.

## Footnotes

1 The portrayed purpose of scientific work as solely communal or agentic varied by publication: NYT included communal more than agentic purpose (*Z* = 2.92, *p* = 0.004), and TS profiles included agentic more than communal purpose (*Z* = –7.39, *p* < 0.001).

## References

[B1] Azvolinsky A. (2015). Of Cells and Limits. *The Scientist Magazine*, March 1.

[B2] Azvolinsky A. (2016). Guts and Glory. *The Scientist Magazine*, April 1.

[B3] Banchefsky S. Westfall J. Park B. Judd C. M. (2016). But you don’t look like a scientist!: women scientists with feminine appearance are deemed less likely to be scientists. *Sex Roles* 75 95–109. 10.1007/s11199-016-0586-1

[B4] Barth J. M. Kim H. Eno C. A. Guadagno R. E. (2018). Matching abilities to careers for others and self: do gender stereotypes matter to students in advanced math and science classes? *Sex Roles* 79 83–97. 10.1007/s11199-017-0857-5

[B5] Bath P. Brenneis J. Burrows L. Eisenstaedt A. Gerdts J. Kilian H. (1961). *U.S. Scientists: Men of the year.* New York, NY: TIME Magazine.

[B6] Benson-Greenwald T. M. Joshi M. P. Diekman A. B. (2021a). *Understanding the Impact of Ecologically Valid Portrayals of Scientists*. Bloomington: Indiana University, Unpublished data.

[B7] Benson-Greenwald T. M. Trujillo A. White A. D. Diekman A. B. (2021b). Science for others or the self? Presumed motives for science shape public trust in science. *Pers. Soc. Psychol. Bull.* 10.1177/01461672211064456 34964420

[B8] Biddle B. J. (1986). Recent developments in role theory. *Annu. Rev. Sociol.* 12 67–92. 10.1146/annurev.so.12.080186.000435

[B9] Blackwell K. L. Trzesniewski K. H. Dweck C. S. (2007). Implicit theories of intelligence predict achievement across an adolescent transition: a longitudinal study and an intervention in child. *Child Dev.* 78 246–263. 10.1111/j.1467-8624.2007.00995.x 17328703

[B10] Boucher K. L. Fuesting M. A. Diekman A. B. Murphy M. C. (2017). Can I work with and help others in this field? How communal goals influence interest and participation in STEM fields. *Front. Psychol.* 8:901. 10.3389/fpsyg.2017.00901 28620330 PMC5450619

[B11] Brescoll V. LaFrance M. (2011). The correlates and consequences of newspaper reports of research on sex differences. *Psychol. Sci.* 15 515–520. 10.1111/j.0956-7976.2004.00712.x 15270995

[B12] Broad W. J. (2014). Seeker, Doer, Giver, Ponderer. *The New York Times*, July 7.

[B13] Brown E. R. Steinberg M. Lu Y. Diekman A. B. (2018). Is the lone scientist an American dream? Perceived communal opportunities in STEM offer a pathway to closing U.S.–Asia gaps in interest and positivity. *Soc. Psychol. Pers. Sci.* 9 11–23. 10.1177/1948550617703173

[B14] Bruce T. (2016). New rules for new times: sportswomen and media representation in the third wave. *Sex Roles* 74 361–376. 10.1007/s11199-015-0497-6

[B15] Bybee R. W. (2010). Advancing STEM education: a 2020 vision. *Technol. Eng. Teach.* 70 30–35.

[B16] Carli L. L. Alawa L. Lee Y. Zhao B. Kim E. (2016). Stereotypes about gender and science: women ≠ scientists. *Psychol. Women Q.* 40 244–260. 10.1177/0361684315622645

[B17] Cheryan S. Plaut V. C. Handron C. Hudson L. (2013). The stereotypical computer scientist: gendered media representations as a barrier to inclusion for women. *Sex Roles* 69 58–71. 10.1007/s11199-013-0296-x

[B18] Chimba M. Kitzinger J. (2010). Bimbo or boffin? Women in science: an analysis of media representations and how female scientists negotiate cultural contradictions. *Public Understand. Sci.* 19 609–624. 10.1177/0963662510377233 21553601

[B19] Clark E. K. Fuesting M. A. Diekman A. B. (2016). Enhancing interest in science: exemplars as cues to communal affordances of science. *J. Appl. Soc. Psychol.* 46 641–654. 10.1111/jasp.12392

[B20] Clark F. Illman D. L. (2006b). Portrayals of engineers in “science times.”. *IEEE Technol. Soc. Magazine* 25 12–21. 10.1109/MTAS.2006.1607718

[B21] Clark F. Illman D. L. (2006a). A Longitudinal Study of the New York times science times section. *Sci. Commun.* 27 496–513. 10.1177/1075547006288010

[B22] Diekman A. B. Brown E. R. Johnston A. M. Clark E. K. (2010). Seeking congruity between goals and roles: a new look at why women opt out of science, technology, engineering, and mathematics careers. *Psychol. Sci.* 21 1051–1057. 10.1177/0956797610377342 20631322

[B23] Diekman A. B. Clark E. K. Johnston A. M. Brown E. R. Steinberg M. (2011). Malleability in communal goals and beliefs influences attraction to STEM careers. *J. Pers. Soc. Psychol.* 101 902–918. 21859224 10.1037/a0025199

[B24] Diekman A. B. Joshi M. P. Benson-Greenwald T. M. (2020). “Goal congruity theory: navigating the social structure to fulfill goals,” in *Advances in Experimental Social Psychology*, Vol. 62 ed. Gawronski B. (Cambridge, MA: Academic Press), 189–244.

[B25] Diekman A. B. Murnen S. K. (2004). Learning to be little women and little men: the inequitable gender equality of nonsexist children’s literature. *Sex Roles* 50 373–385.

[B26] Diekman A. B. Schneider M. C. (2010). A social role theory perspective on gender gaps in political attitudes. *Psychol. Women Q.* 34 486–497. 10.1111/j.1471-6402.2010.01598.x

[B27] Diekman A. B. Steinberg M. Brown E. R. Belanger A. L. Clark E. K. (2017). A goal congruity model of role entry, engagement, and exit: understanding communal goal processes in STEM gender gaps. *Pers. Soc. Psychol. Rev.* 21 142–175. 10.1177/1088868316642141 27052431

[B28] Donnelly K. Twenge J. M. (2017). Masculine and feminine traits on the bem sex-role inventory, 1993–2012: a cross-temporal meta-analysis. *Sex Roles* 76 556–565. 10.1007/s11199-016-0625-y

[B29] Dweck C. S. (ed.) (1999). “Essays in social psychology,” in *Self Theories: Their Role in Motivation, Personality, and Development.* (Hove: Psychology Press).

[B30] Eagly A. H. Nater C. Miller D. I. Kaufmann M. Sczesny S. (2019). Gender stereotypes have changed: a cross-temporal meta-analysis of U.S. public opinion polls from 1946 to 2018. *Am. Psychol.* 75 301–315. 10.1037/amp0000494 31318237

[B31] Eagly A. H. Wood W. (2011). “Social role theory,” in *Handbook of Theories in Social Psychology*, Vol. 2 eds Van Lange P. A. M. Kruglanski A. W. Higgins E. T. (Thousand Oaks, CA: Sage), 458–476.

[B32] Emerson K. T. U. Murphy M. C. (2015). A company I can trust? Organizational lay theories moderate stereotype threat for women. *Pers. Soc. Psychol. Bull.* 41 295–307. 10.1177/0146167214564969 25534242

[B33] Ficko Z. Koo K. Hyams E. S. (2017). High tech or high risk? An analysis of media reports about robotic surgery. *J. Robotic Surg.* 11 211–216. 10.1007/s11701-016-0647-z 27778227

[B34] Fuesting M. A. Diekman A. B. (2017). Not by success alone: role models provide pathways to communal opportunities in STEM. *Pers. Soc. Psychol. Bull.* 43 163–176. 10.1177/0146167216678857 27932632

[B35] Fürsich E. Lester E. P. (1996). Science journalism under scrutiny: a textual analysis of “science times.”. *Crit. Stud. Mass Commun.* 13 24–43. 10.1080/15295039609366958

[B36] Good C. Rattan A. Dweck C. S. (2012). Why do women opt out? Sense of belonging and women’s representation in mathematics. *J. Pers. Soc. Psychol.* 102 700–717. 10.1037/a0026659 22288527

[B37] Han S. Shavitt S. (1994). Persuasion and culture: advertising appeals in individualistic and collectivistic societies. *J. Exp. Soc. Psychol.* 30 326–350.

[B38] Koenig A. M. Eagly A. H. (2014). Evidence for the social role theory of stereotype content: observations of groups’ roles shape stereotypes. *J. Pers. Soc. Psychol.* 107 371–392. 10.1037/a0037215 25133722

[B39] Leslie S.-J. Cimpian A. Meyer M. Freeland E. (2015). Expectations of brilliance underlie gender distributions across academic disciplines. *Science* 347 262–265. 10.1126/science.1261375 25593183

[B40] Lievrouw L. A. (1990). Communication and the social representation of scientific knowledge. *Crit. Stud. Mass Commun.* 7 1–10.

[B41] Lievrouw L. A. (1992). Communication, representation, and scientific knowledge: a conceptual framework and case study. *Knowl. Policy* 5 6–28. 10.1007/BF02692789

[B42] Markus H. R. Kitayama S. (1991). Culture and the self: implications for cognition, emotion, and motivation. *Psychol. Rev.* 98 224–253. 10.1037/0033-295X.98.2.224

[B43] Markus H. R. Kitayama S. (2010). Cultures and selves: a cycle of mutual constitution. *Perspect. Psychol. Sci.* 5 420–430. 10.1177/1745691610375557 26162188

[B44] McFarland J. Hussar B. Wang X. Zhang J. Wang K. Rathbun A. (2018). *The Condition of Education 2018 (NCES 2018-144).* Washington, DC: U.S. Department of Education.

[B45] Mitchell M. McKinnon M. (2019). ‘Human’ or ‘objective’ faces of science? Gender stereotypes and the representation of scientists in the media. *Public Understand. Sci.* 28 177–190. 10.1177/0963662518801257 30247096

[B46] Moskowitz D. S. Suh E. J. Desaulniers J. (1994). Situational influences on gender differences in agency and communion. *J. Pers. Soc. Psychol.* 66 753–761. 10.1037/0022-3514.66.4.753 8189350

[B47] Murphy M. C. Dweck C. S. (2010). A culture of genius: how an organization’s lay theory shapes people’s cognition, affect, and behavior. *Pers. Soc. Psychol. Bull.* 36 283–296. 10.1177/0146167209347380 19826076

[B48] National Science Board (2018). *Science and Engineering Indicators 2018.* Alexandria: National Science Board.

[B49] Orthia L. A. Morgain R. (2016). The gendered culture of scientific competence: a study of scientist characters in Doctor Who 1963–2013. *Sex Roles* 75 79–94. 10.1007/s11199-016-0597-y

[B50] Pennebaker J. W. Booth R. J. Boyd R. L. Francis M. E. (2015a). *Linguistic Inquiry and Word Count: LIWC2015.* Austin, TX: Pennebaker Conglomerates.

[B51] Pennebaker J. W. Boyd R. L. Jordan K. Blackburn K. (2015b). *The Development and Psychometric Properties of LIWC2015.* Austin, TX: University of Texas at Austin.

[B52] Pew Research Center (2012). *Section 4: Demographics and Political Views of News Audiences.* Washington, DC: Pew Research Center.

[B53] Pienta R. S. Smith M. (2012). “Women on the margins: the politics of gender in the language and content of science textbooks,” in *The New Politics of the Textbook: Problematizing the Portrayal of Marginalized Groups in Textbooks*, eds Hickman H. Porfilio B. J. (Cham: Springer), 49–68.

[B54] Pietraszkiewicz A. Formanowicz M. Sendén M. G. Boyd R. L. Sikström S. Sczesny S. (2019). The big two dictionaries: capturing agency and communion in natural language. *Eur. J. Soc. Psychol.* 49 871–887. 10.1002/ejsp.2561

[B55] Pietri E. S. Johnson I. R. Majid S. Chu C. (2020). Seeing what’s possible: videos are more effective than written portrayals for enhancing the relatability of scientists and promoting black female students’ interest in STEM. *Sex Roles* 84 14–33. 10.1007/s11199-020-01153-x

[B56] Previs K. K. (2016). Gender and race representations of scientists in Highlights for Children: a content analysis. *Sci. Commun.* 38 303–327. 10.1177/1075547016642248

[B57] Roter D. L. Hall J. A. (1998). Why physician gender matters in shaping the physician-patient relationship. *J. Womens Health* 7 1093–1097. 9861586 10.1089/jwh.1998.7.1093

[B58] Roter D. L. Hall J. A. Aoki Y. (2002). Physician gender effects in medical communication: a meta-analytic review. *J. Am. Med. Assoc.* 288 756–764. 10.1001/jama.288.6.756 12169083

[B59] Schneider M. C. Holman M. R. Diekman A. B. McAndrew T. (2016). Power, conflict, and community: how gendered views of political power influence women’s political ambition. *Polit. Psychol.* 37 515–531. 10.1111/pops.12268

[B60] Scudellari M. (2013a). Up, up, and array. *The Scientist Magazine*, April 1.

[B61] Scudellari M. (2013b). Waste-management consultant. *The Scientist Magazine*, November 1.

[B62] Shachar O. (2000). Spotlighting women scientists in the press: tokenism in science journalism. *Public Understand. Sci.* 9 347–358. 10.1088/0963-6625/9/4/301

[B63] Smith J. L. Cech E. Metz A. Huntoon M. Moyer C. (2014). Giving back or giving up: native American student experiences in science and engineering. *Cult. Divers. Ethnic. Minor. Psychol.* 20 413–429. 10.1037/a0036945 25045952

[B64] Smith J. L. Lewis K. L. Hawthorne L. Hodges S. D. (2013). When trying hard isn’t natural: women’s belonging with and motivation for male-dominated STEM fields as a function of effort expenditure concerns. *Pers. Soc. Psychol. Bull.* 39 131–143. 10.1177/0146167212468332 23187722

[B65] Snibbe A. C. Markus H. R. (2005). You can’t always get what you want: educational attainment, agency, and choice. *J. Pers. Soc. Psychol.* 88 703–720. 10.1037/0022-3514.88.4.703 15796669

[B66] Steinberg M. Diekman A. B. (2018). Considering “why” to engage in STEM activities elevates communal content of STEM affordances. *J. Exp. Soc. Psychol.* 75 107–114. 10.1016/j.jesp.2017.10.010

[B67] Steinke J. (2005). Cultural representations of gender and science: portrayals of female scientists and engineers in popular films. *Sci. Commun.* 27 27–63. 10.1177/1075547005278610

[B68] Steinke J. (2013). “Portrayals of female scientists in the mass media,” in *The International Encyclopedia of Media Studies*, eds Valdivia A. Mazzarella S. R. (Hoboken, NJ: Blackwell Publishing Ltd), 1–18.

[B69] Steinke J. Tavarez P. M. P. (2018). Cultural representations of gender and STEM: portrayals of female STEM characters in popular films 2002-2014. *Int. J. Gender Sci. Technol.* 9 244–277.

[B70] Stout J. G. Grunberg V. A. Ito T. A. (2016). Gender roles and stereotypes about science careers help explain women and men’s science pursuits. *Sex Roles* 75 490–499. 10.1007/s11199-016-0647-5

[B71] Subramaniam R. (2014). “Public education about science in singapore: the role of science journalism via newspapers,” in *Inquiry into the Singapore Science Classroom*, eds Tan A. L. Poon C. L. Lim S. (Berlin: Springer), 273–296.

[B72] Swers M. L. (2013). *Women in the Club: Gender and Policy Making in the Senate.* Chicago, IL: University of Chicago Press.

[B73] The Scientist (2021). *Our Audience.* New York, NY: The Scientist.

[B74] Triandis H. C. (1989). The self and social behavior in differing cultural contexts. *Psychol. Rev.* 96 506–520. 10.1037/0033-295X.96.3.506

[B75] Trope Y. Liberman N. (2010). Construal-level theory of psychological distance. *Psychol. Rev.* 117 440–463. 10.1037/a0018963 20438233 PMC3152826

[B76] Vence T. (2015). Engineer of Change. *The Scientist Magazine*, February 1.

[B77] Yeager D. S. Henderson M. D. Paunesku D. Walton G. M. D’Mello S. Spitzer B. J. (2014). Boring but important: a self-transcendent purpose for learning fosters academic self-regulation. *J. Pers. Soc. Psychol.* 107 559–580. 10.1037/a0037637 25222648 PMC4643833

